# Longitudinal frailty trajectories and risk of incident chronic liver disease among middle-aged and older Chinese adults: the mediating role of depressive symptoms

**DOI:** 10.21203/rs.3.rs-10004954/v1

**Published:** 2026-07-10

**Authors:** Yingxin Liu, Lanyu Chen

**Affiliations:** 1 Department of Infectious Diseases, Guang ’anmen Hospital of China Academy of Chinese Medical Sciences, Beijing, China

**Keywords:** Chronic liver disease, Frailty index, Depressive symptoms, Trajectory, CHARLS

## Abstract

**Background:**

Chronic liver disease imposes a substantial global health burden, a challenge further compounded by population aging. Prior studies have predominantly relied on frailty index assessments at a single baseline time point, the study is the first to apply group-based trajectory modeling to construct longitudinal frailty index trajectories, with the aim of elucidating their independent association with incident CLD among middle-aged and older Chinese adults and further exploring the potential mediating role of depressive symptoms.

**Methods:**

To examine the association between frailty and incident chronic liver disease (CLD) and the mediating role of depressive symptoms, we enrolled 7,363 participants aged ≥45 years from the China Health and Retirement Longitudinal Study (CHARLS) and performed Cox proportional hazards regression and mediation analyses.

**Results:**

Over 5 years, 385 participants developed chronic liver disease. After adjustment for confounders, individuals in the frailty-worsening trajectory group had a 48% increased risk of incident chronic liver disease (CLD) (HR = 1.48, 95% CI: 1.16–1.90). Depressive symptoms mediated 25.6% of the total effect, which was statistically significant yet relatively modest in magnitude

**Conclusion:**

Among middle-aged and older Chinese adults, a frailty-worsening trajectory was significantly associated with the risk of incident chronic liver disease, and depressive symptoms partially mediated this association. This finding suggests that regular comprehensive assessment of both physical function and mental health in older adults may represent an important strategy for CLD prevention.

## Introduction

Chronic liver disease (CLD) encompasses a spectrum of conditions, including chronic viral hepatitis, autoimmune hepatitis, nonalcoholic fatty liver disease (NAFLD), and cirrhosis. Globally, liver disease and its complications account for approximately 2 million deaths annually, representing 4% of total global mortality, with cirrhosis and its complications being responsible for the majority of these deaths[1,2]. The prevalence of CLD in China is steadily increasing, currently affecting over 20% of the population. Viral hepatitis and metabolic-associated fatty liver disease (MAFLD) are the two major etiologies, while cirrhosis constitutes the critical pathological substrate for end-stage liver disease[3,4]. Therefore, early risk stratification and the formulation of effective preventive strategies for CLD in middle-aged and older populations are of considerable public health significance, as they can reduce the burden on healthcare systems, lower mortality from CLD, and improve the quality of life of older adults

Frailty is a clinical syndrome characterized by decline across multiple physiological systems and heightened vulnerability to stressors[5]. Prior studies have shown that frailty is an independent risk factor for adverse clinical outcomes in patients with cirrhosis and end-stage liver disease, significantly associated with pre- and post-liver transplant mortality, prolonged hospitalization, infection, and disability[6–8]. Large-scale prospective cohort studies have further confirmed that baseline frailty independently predicts the development of nonalcoholic fatty liver disease (NAFLD) and the progression of liver disease[9]. Among middle-aged and older Chinese adults, frailty is also significantly associated with the risk of incident CLD. Collectively, these findings indicate that frailty is a valuable clinical indicator for both the early identification of liver disease risk and the prognostic assessment of end-stage liver disease[10]. However, most existing studies rely on single baseline measurements and have not fully captured the dynamic nature of frailty.

Notably, frailty involves not only declines in physical function but also profound changes in psychological dimensions. Mendelian randomization studies have established a bidirectional causal relationship between frailty and depression, showing that genetically predicted higher frailty index is significantly associated with an increased risk of depression, and vice versa[11]. This finding suggests that frailty and depression may share common biological underpinnings, such as systemic inflammation and structural brain degeneration[12]. Meanwhile, mounting evidence indicates that depressive symptoms are not only positively associated with the risk of developing CLD but may also directly exacerbate liver injury through unhealthy lifestyle behaviors and neuroendocrine pathways[13,14]. Against the background, depressive symptoms are regarded as an important psychological pathway linking physical functional decline to liver disease. However, no study has yet systematically examined this longitudinal mediation mechanism.

Therefore, using longitudinal data from the China Health and Retirement Longitudinal Study (CHARLS, 2011–2020), this study constructed frailty index trajectories among community-dwelling middle-aged and older adults to elucidate the independent association between dynamic frailty worsening and incident CLD risk, and to further explore the potential mediating role of depressive symptoms.

## Methods

### Study population

The China Health and Retirement Longitudinal Study (CHARLS) is a nationally representative prospective cohort study of adults aged 45 years and older in China, systematically collecting social, economic, and health-related data. The survey employs a multistage stratified probability-proportional-to-size (PPS) sampling design, covering 150 counties/districts and 450 villages/communities across 28 provinces nationwide. The baseline survey was conducted in 2011, with biennial follow-up waves thereafter. The study was approved by the Biomedical Ethics Review Committee of Peking University (approval number: IRB00001052–11015), and all participants provided written informed consent. Further details are available on the official website: http://charls.pku.edu.cn/en[15].

**Figure 1:**
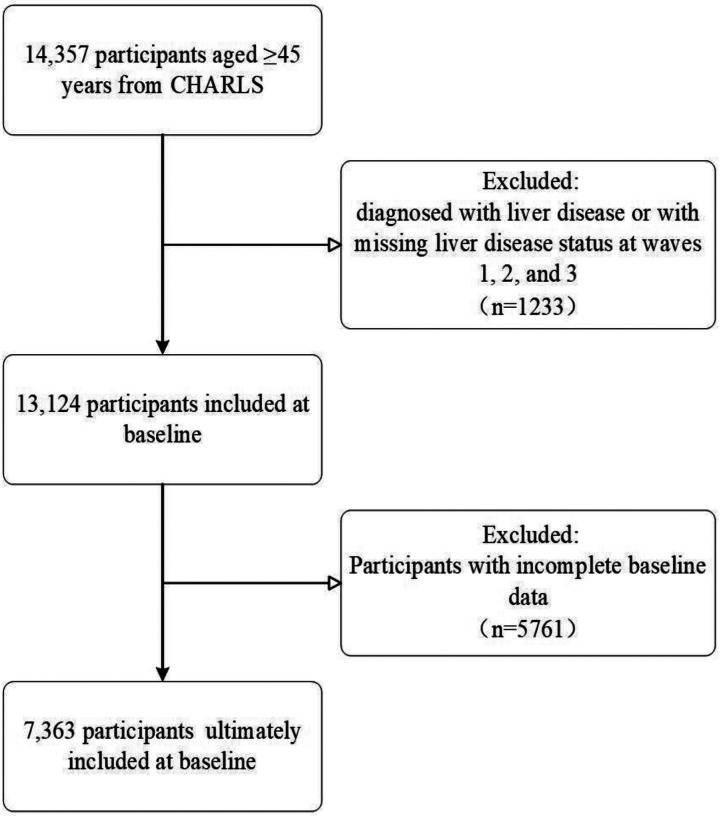


This study used data from the China Health and Retirement Longitudinal Study (CHARLS) spanning 2011 to 2020. The baseline dataset comprised 14,357 participants aged ≥45 years. We excluded those diagnosed with liver disease or with missing liver disease status from 2011 to 2015 (n = 1,233), and subsequently excluded participants with incomplete baseline data (n = 5,761). Ultimately, 7,363 eligible participants were retained for analysis. (Figure 1)

### Frailty index

The FI was developed according to established procedures and principles outlined by Searle SD[16,17]. The frailty index (FI) was constructed using 32 CHARLS items covering morbidity, hearing/visual impairment, ADL/IADL disability, physical functioning, self-rated health, and depression. Each item was scored 1 (deficit) or 0 (no deficit)[18]. In accordance with prior studies, the FI was calculated by summing the 32 deficit scores (range 0–32) and dividing each participant’s total by 32. This yielded a continuous frailty index score bounded between 0 and 1, with higher values indicating greater frailty.

### Depressive symptoms

Depressive symptoms were assessed using the Center for Epidemiologic Studies Depression Scale (CES-D), a validated tool designed to evaluate the frequency of depressive symptoms experienced over the past week. Responses for each item were classified into four levels: “Rarely or none,” “Some or a little,” “Occasionally or moderate,” and “Most or all the time.” The CES-D includes 10 items: 1. being bothered by things that usually do not trouble you, 2. difficulty keeping your mind focused, 3. feeling depressed, 4. feeling everything you do is an effort, 5. feeling hopeful about the future, 6. feeling fearful, 7. restless sleep, 8. feeling happy, 9. feeling lonely, and 10. feeling like you cannot move forward. Before computing the total score, items 5 and 8 were reverse-coded. Each item was scored 0, 1, 2, or 3. The CES-D total score ranges from 0 to 30, with higher scores indicating greater severity of depressive symptoms[19].

### Chronic liver disease

Chronic liver disease (CLD) was diagnosed by a physician and reported by the patient during each visit. Based on the questionnaire design of the CHARLS, CLD in this study was defined as encompassing viral hepatitis, autoimmune hepatitis, primary biliary cholangitis, and primary sclerosing cholangitis, but explicitly excluding fatty liver disease and hepatic malignancies. To reduce potential recall bias, participants were additionally asked whether they were “currently receiving any treatment for chronic liver disease or its complications, such as traditional Chinese medicine, Western medicine, or other therapies.” The responses to these two questions were combined to classify CLD status as “Yes” or “No”[20].

### Assessment of covariates

Covariates included information on participants’ sociodemographic characteristics, lifestyle behaviors, and history of chronic diseases. Sociodemographic characteristics comprised age, sex/gender (female or male), educational attainment (Less than lower secondary education or secondary or above), place of residence (City/town or Village), and marital status (Married or never-married/separated/widowed). Lifestyle behaviors included smoking status (never/former/current) and drinking status (never/Drink but less than once a month/Drink but more than once a month). Body mass index (BMI) was obtained from physical examination and was calculated as weight in kilograms divided by the square of height in meters[21].History of chronic diseases included hypertension, diabetes, dyslipidemia, heart disease and cancer (all coded as Yes or No).

### Statistical analysis

In the study, Group-based trajectory modeling (GBTM) was employed to identify distinct trajectories of the frailty index from 2011 to 2015. First, baseline characteristics were summarized by trajectory groups of the frailty index. Continuous variables were expressed as mean ± standard deviation, categorical variables were reported as frequencies and percentages. Between-group differences were assessed using one-way analysis of variance (ANOVA) for continuous variables, the Kruskal–Wallis rank-sum test for the CES-D score, and the chi-squared test (or Fisher's exact test when cell frequencies were less than 5) for categorical variables.

Second, with the robust-stable trajectory group serving as the reference, Cox proportional hazards regression models were used to estimate the associations between frailty index trajectory groups and incident CLD, with results expressed as hazard ratios (HRs) and 95% confidence intervals (CIs). Kaplan–Meier cumulative incidence curves were plotted to depict the temporal trends in CLD cumulative incidence for the two groups, and the log-rank test was employed to compare between-group differences. Model 1 was adjusted for age, sex, education, marital status, and place of residence. Model 2 was further adjusted for smoking status, alcohol consumption, and body mass index (BMI) in addition to the covariates in Model 1. Model 3 was further adjusted for history of chronic diseases (hypertension, diabetes, heart disease, cancer, and dyslipidemia) on the basis of Model 2. Model 4 was additionally adjusted for depressive symptom score as a mediator

The mediation model proposed by Baron and Kenny (1986) was used to assess the mediating effect of depressive symptoms on the association between the frailty index trajectory group and incident CLD. The indirect, direct, and total effects were calculated by integrating the mediation and outcome models, with adjustment for all covariates in Model 3. The specific steps of the mediation analysis were as follows: (1) estimating the total effect of the frailty index trajectory group on incident CLD; (2) examining the effect of the frailty index trajectory group on the CES-D score; and (3) evaluating the direct effect of the frailty index trajectory group on incident CLD and the indirect effect through the CES-D score. The proportion mediated was calculated as [indirect effect / (indirect effect + direct effect)] × 100%[22].

Additionally, participants with cancer in 2015 were excluded to eliminate potential confounding from cancer-related pain and wasting. Subgroup and interaction analyses were then performed, stratified by age, sex, place of residence, marital status, education level, smoking status, alcohol consumption, BMI, hypertension, dyslipidemia, heart disease and diabetes.

## Results

**Figure 2:**
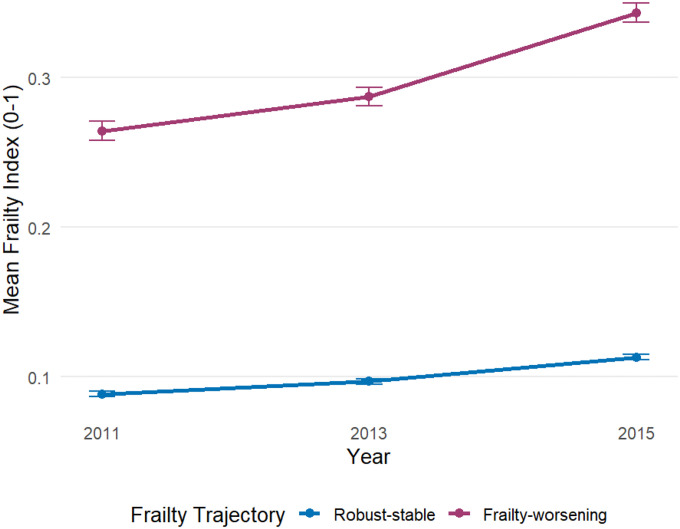


After applying the inclusion and exclusion criteria, a total of 7,363 participants were included in the final analysis, of whom 385 developed incident CLD during follow-up. The mean age of the cohort was 61.51 ±8.64 years, and 54.87% were female. Two distinct frailty index trajectory groups were identified: a robust-stable trajectory (n = 5997, 81.45%) and a frailty-worsening trajectory (n = 1,366, 18.55%) (Figure 2). Baseline characteristics of the participants included in the analysis are presented in Table 1. There were statistically significant differences between the two frailty index trajectory groups in the distribution of age, sex, place of residence, marital status, education level, smoking status, alcohol consumption, hypertension, dyslipidemia, heart disease, cancer, diabetes, and CES-D score (Table 1).

**Figure 3:**
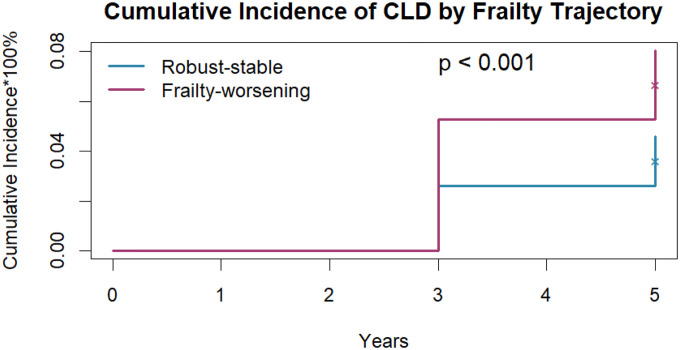


Figure 3 presents the Kaplan–Meier cumulative incidence curves for CLD stratified by frailty index trajectory group. The two curves were clearly separated by 2018, and the separation persisted through the subsequent two years of follow-up. At the end of the 5-year observation period, the cumulative incidence of CLD was 8.1% in the frailty-worsening group versus 4.6% in the robust-stable group, with the difference being statistically significant (log-rank test, P < 0.001).(Table 2) The frailty index was positively associated with the risk of CLD across all three models (Model 1, Model 2, and Model 3). In the fully adjusted model (Model 3), the frailty-worsening group had a 48% increased risk of CLD (HR = 1.48, 95% CI 1.16–1.90, P < 0.05). Similarly, higher CES-D scores were significantly associated with an elevated risk of CLD. After further adjustment for depressive symptoms (Model 4), the association between the frailty index and CLD was attenuated (P < 0.05). (Table 3)

### Mediation analysis

We then examined the mediating effect of depressive symptoms on the association between the frailty index and incident CLD(Figure 4). First, after controlling for all covariates, linear regression was performed with the frailty index as the independent variable and depressive symptoms (CES-D score) as the dependent variable. The results showed that the frailty index was positively associated with depressive symptoms (P < 0.001). Second, Cox regression was performed with the CES-D score as the independent variable and incident CLD as the dependent variable. Higher CES-D scores were associated with an increased risk of CLD (P < 0.05). The mediation analysis revealed that depressive symptoms partially mediated the association between the frailty index and incident CLD, with a mediated proportion of 25.6% (indirect effect = 0.105; 95% CI, 0.005–0.195).

### Subgroups and sensitivity analyses

Sensitivity analyses excluding participants with cancer confirmed the robustness of the results. (Table 4). Subgroups analyses stratified by age, sex, place of residence, marital status, education level, smoking status, alcohol consumption, and history of chronic diseases were conducted to assess whether the association between the frailty index and incident chronic liver disease (CLD) was consistent across population subgroups. As shown in Fig. 2, the frailty-worsening trajectory group showed a trend toward an increased risk of incident CLD in all subgroups. However, P values were > 0.05 in some subgroups, possibly because the small number of events in these subgroups resulted in insufficient statistical power. This does not necessarily indicate that the frailty index has no detrimental effect in these populations. In addition, formal tests for interaction could not be reliably performed owing to the limited number of events in certain strata. (Figure 5)

## Discussion

Prior studies have linked frailty with chronic liver disease. For example, the UK Biobank longitudinal study that grouped participants by the Fried physical frailty phenotype and the Rockwood frailty index reported a 2- to 4-fold higher risk of liver disease in frail versus non-frail individuals[23]. Furthermore, a prospective study of 5,506 middle-aged and older Chinese adults found that individuals with frailty alone had a 66% increased risk of incident digestive diseases; however, the risk specific to chronic liver disease was not reported in that study[24]. Other studies have shown that baseline frailty is an independent predictor of incident CLD, conferring a 30%–70% increased risk[10]. Building on baseline frailty research, this study leveraged three repeated frailty index assessments to construct dynamic frailty trajectories, thereby refining risk stratification for incident chronic liver disease and uncovering a partial mediating role of depressive symptoms.

The results showed that, compared with participants in the robust-stable trajectory group, those in the frailty-worsening trajectory group had a hazard ratio (HR) of 1.48 (95% confidence interval [CI], 1.16–1.90; p < 0.05) for incident chronic liver disease (CLD). Unlike a single baseline frailty measurement, the frailty-worsening trajectory captures a progressive rise in the frailty index over five years, reflecting a continual loss of physiological reserve. This indicates that the development of CLD is not merely related to frailty at one time point but is more strongly associated with sustained physical functional decline. Meanwhile, the indirect effect of depressive symptoms on the association between the frailty-worsening trajectory and incident CLD was 0.105(bootstrap 95% CI, 0.005–0.195), accounting for approximately 25.6% of the total effect, suggesting that psychological distress constitutes an important intermediate pathway through which worsening frailty contributes to liver injury.

The biological mechanisms linking frailty to incident CLD are complex and primarily involve three major pathways. The first is immune dysfunction and persistent inflammation. Given that viral hepatitis remains the predominant cause of CLD among middle-aged and older Chinese adults, frailty-related exacerbation of T-cell dysfunction may compromise immunological control of viral replication, thereby accelerating the transition from asymptomatic carriage to active disease[25,26]. Meanwhile, the cumulative inflammatory burden persistently stimulates hepatic Kupffer cells and stellate cells, leading to the sustained release of inflammatory mediators that exacerbate fibrogenesis. The second pathway involves metabolic dysfunction. Reduced muscle mass—a hallmark feature of frailty—has been significantly associated with the risk of developing nonalcoholic fatty liver disease (NAFLD). Loss of skeletal muscle mass leads to systemic insulin resistance, mitochondrial dysfunction, and dysregulated myokine secretion, which in turn promote hepatic fat accumulation and oxidative stress. This lipotoxicity further exacerbates liver inflammation and apoptosis, ultimately contributing to progressive hepatic fibrosis[28,29].The third pathway involves gut dysbiosis. Evidence suggests that frailty is associated with an imbalance in intestinal T-cell subsets and disruption of the gut barrier[30]. Gut dysbiosis leads to an increased endotoxin load, which activates the Toll-like receptor 4 pathway, triggering chronic inflammation and exacerbating liver injury. Concurrently, portal hypertension and altered bile acid secretion compromise the intestinal barrier, promoting the translocation of bacteria and their products. The resulting elevated endotoxin levels activate hepatic stellate cells via the TLR4 pathway, further aggravating hepatocellular damage and structural deterioration of the liver[31].

Sustained physical functional decline is often accompanied by reduced mobility, social withdrawal, and loss of independence—changes that may induce or exacerbate depressive symptoms. Meanwhile, depressive symptoms are frequently associated with unhealthy lifestyle behaviors such as smoking, alcohol consumption, high-calorie diets, and physical inactivity, all of which are established risk factors for CLD[32,33]. Second, chronic psychological stress induced by depression can lead to persistent excitation of the sympathetic nervous system and hyperactivation of the hypothalamic–pituitary–adrenal (HPA) axis, resulting in sustained elevations of cortisol and catecholamine neurotransmitters that, in turn, exacerbate intrahepatic inflammation and promote fibrosis and even cirrhosis. Furthermore, the biological mechanisms underlying depressive symptoms and those linking frailty to CLD overlap, potentially inflicting superimposed liver damage through shared inflammatory–metabolic pathways[19,34,35].

In summary, the aforementioned mechanisms not only directly damage the liver through immune, metabolic, and gut pathways but also act synergistically with depressive symptoms triggered by sustained functional decline, collectively promoting the onset and progression of CLD.

Given that China confronts the dual challenges of rapid population aging and a heavy liver disease burden, the present findings carry clear practical significance and clinical value. First, the FI, constructed from questionnaire-based information and simple physical assessments, may serve as a valuable complement to existing CLD risk-stratification tools by enabling continuous, dynamic monitoring to identify vulnerable individuals at high risk even before CLD onset. Second, for older adults with a persistently rising FI, mental health screening should be proactively performed alongside routine physical function interventions. Finally, systematic frailty assessment and psychological evaluation should be integrated into longitudinal disease management to reduce the risk of incident CLD through early identification and management of frailty and depressive symptoms. At the physical level, regular monitoring of functional status changes is recommended, with timely nutritional support and exercise prescription for those showing sustained deterioration, so as to delay further loss of physiological reserve. At the psychological level, mood screening should be incorporated into routine geriatric assessment, and early intervention for depressive symptoms should be implemented to interrupt their indirect liver damage through neuroendocrine and behavioral pathways.

Several limitations should be acknowledged. First, CLD status and frailty-related variables in CHARLS relied on self-reported data, which precluded precise etiological classification and disease staging and may have introduced misclassification and recall bias. Second, owing to insufficient event numbers within subgroups, formal interaction tests could not be reliably performed to determine whether the between-group differences in the effects of worsening frailty were statistically significant. Finally, CHARLS primarily covers community-dwelling middle-aged and older adults, with limited coverage of individuals residing in nursing homes or long-term care facilities. Given that this population tends to have more severe frailty and a potentially higher risk of CLD, our findings may underestimate the true impact of worsening frailty on CLD.

## Conclusions

This study demonstrates that frailty index trajectories are closely associated with the risk of incident chronic liver disease (CLD) among middle-aged and older Chinese adults. Individuals in the frailty-worsening trajectory group had a significantly higher risk of developing CLD than those in the robust-stable trajectory group, suggesting that dynamic, longitudinal functional decline better captures changes in susceptibility to liver disease than a single static assessment. Notably, this association remained independent and significant after full adjustment for traditional risk factors, underscoring the potential of the frailty-worsening trajectory as an early warning indicator for CLD. Longitudinal trajectory-based frailty assessment may help identify high-risk individuals more precisely and offer a novel dynamic stratification framework for the early prevention and targeted intervention of CLD. Furthermore, this study found that depressive symptoms partially mediated the relationship between worsening frailty and CLD risk, indicating that psychological distress constitutes an important intermediate pathway linking functional decline to liver damage. Future efforts should prioritize integrating frailty management with mental health care, implementing comprehensive interventions targeting both sustained functional deterioration and mood disorders, so as to mitigate the risk of developing CLD and ultimately improve the long-term prognosis of this vulnerable population.

## Figures and Tables

**Figure 4:**
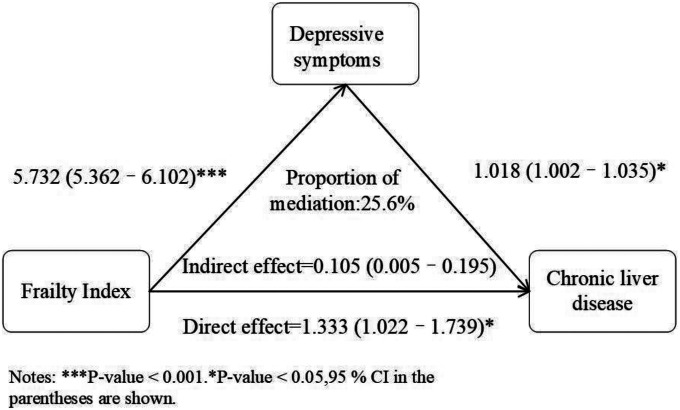


**Figure 4:**
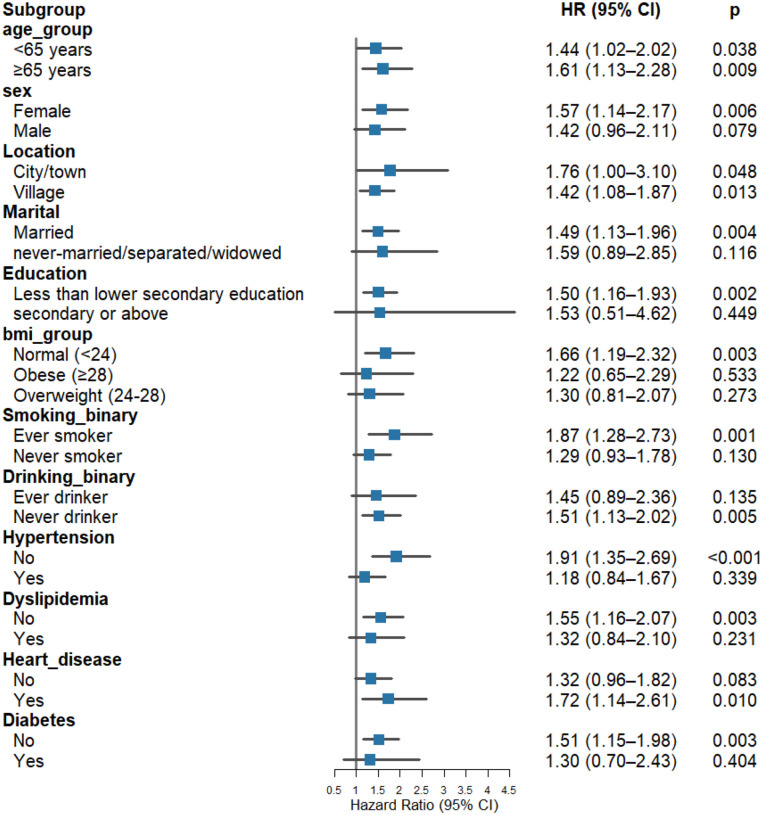


**Table 1:** Baseline characteristics of the study population by frailty index trajectories

Variable	Overall	Robust-stable	Frailty-worsening	p
	N=7363	N=5997	N=1366	
Age (mean ±SD)	61.51 ±8.64	60.68 ±8.43	65.15 ±8.61	<0.001
BMI (mean ±SD)	24.86 ±32.57	24.75 ±34.65	25.34 ±21.14	0.544
CES-D (mean ±SD)	7.69 ±6.35	6.58 ±5.52	12.56 ±7.37	<0.001
sex, n (%)				<0.001
Female	4040 (54.87%)	3098(51.66%)	942 (68.96%)	
Male	3323 (45.13%)	2899(48.34%)	424 (31.04%)	
Location, n (%)				<0.001
City/town	1215 (16.50%)	1061(17.69%)	154 (11.27%)	
Village	6148 (83.50%)	4936(82.31%)	1212 (88.73%)	
Marital, n (%)				<0.001
Married	6441 (87.48%)	5352(89.24%)	1089 (79.72%)	
never-married/separated/widowed	922 (12.52%)	645(10.76%)	277 (20.28%)	
Education, n (%)				<0.001
Less than lower secondary education	6600 (89.64%)	5285(88.13%)	1315 (96.27%)	
secondary or above	763 (10.36%)	712(11.87%)	51 (3.73%)	
Smoking, n (%)				<0.001
Current smoker	1988 (27.00%)	1725(28.76%)	263 (19.25%)	
Ex-smoker	1155 (15.69%)	926(15.44%)	229 (16.76%)	
Non-smoker	4220 (57.31%)	3346(55.79%)	874 (63.98%)	
Drinking, n (%)				<0.001
Drink but less than once a month	607 (8.24%)	527 (8.79%)	80 (5.86%)	
Drink more than once a month	1873 (25.44%)	1658(27.65%)	215 (15.74%)	
never	4883 (66.32%)	3812(63.57%)	1071 (78.40%)	
Cancer, n (%)				0.009
No	7251 (98.71%)	5917(98.88%)	1334 (97.94%)	
Yes	95 (1.29%)	67(1.12%)	28 (2.06%)	
Hypertension, n (%)				<0.001
No	5000 (67.91%)	4359(72.69%)	641 (46.93%)	
Yes	2363 (32.09%)	1638(27.31%)	725 (53.07%)	
Dyslipidemia, n (%)				<0.001
No	6079 (82.56%)	5081(84.73%)	998 (73.06%)	
Yes	1284 (17.44%)	916 (15.27%)	368 (26.94%)	
Heart disease, n (%)				<0.001
No	6181 (83.95%)	5291(88.23%)	890 (65.15%)	
Yes	1182 (16.05%)	706(11.77%)	476 (34.85%)	
Diabetes, n (%)				<0.001
No	6726 (91.35%)	5594(93.28%)	1132 (82.87%)	
Yes	637 (8.65%)	403 (6.72%)	234 (17.13%)	

**Table 2:** Cumulative CLD incidence (%) by frailty trajectory group and follow up year.

Year	Robust-stable	Frailty-worsening
0	0	0
3	2.60%	5.30%
5	4.60%	8.10%

**Table 3:** Trajectories of frailty index and risk of CLD (Multivariable Cox regression models)

	Robust-stable	Frailty-worsening	
Case, n (%)	275 (4.6)	110 (8.0)	
Model	HR	HR_95CI	p
Unadjusted	1.00 (Ref)	1.79 (1.44–2.23)	<0.001
Model 1	1.00 (Ref)	1.83(1.45–2.31)	<0.001
Model 2	1.00 (Ref)	1.82 (1.45–2.31)	<0.001
Model 3	1.00 (Ref)	1.48 (1.16–1.90)	0.002
Model 4	1.00 (Ref)	1.33 (1.02– 1.73)	0.003

Data are odds ratios (95% CI)

Model 1= age, sex, education, marital status, residence

Model 2=Model 1+ BMI, smoking, drinking

Model 3 =Model 2+ hypertension, diabetes, heart disease, Cancer, dyslipidemia

Model 4=Model 3+CESD-score

**Table 4:** Sensitivity analysis excluding participants with cancer at baseline.

Sensitivity Analysis	HR_95CI	p
Model 3	1.48 (1.16–1.90)	<0.001
Sensitivity Model	1.47 (1.14–1.89)	0.00279

Sensitivity Model=Model 3-Cancer

## Data Availability

More information regarding obtaining data for research use can be found at the CHARLS database (http://charls.pku.edu.cn/en)

## References

[1] Devarbhavi H, Asrani SK, Arab JP, Nartey YA, Pose E, Kamath PS. Global burden of liver disease: 2023 update. J Hepatol. 2023;79(2):516–537. 36990226 10.1016/j.jhep.2023.03.017

[2] Huang DQ, Terrault NA, Tacke F, Global epidemiology of cirrhosis - aetiology, trends and predictions. Nat Rev Gastroenterol Hepatol. 2023;20(6):388–398. 36977794 10.1038/s41575-023-00759-2 PMC10043867

[3] Xiao J, Wang F, Wong NK, Global liver disease burdens and research trends: Analysis from a Chinese perspective. J Hepatol. 2019;71(1):212–221. 30871980 10.1016/j.jhep.2019.03.004

[4] Peng J, Huang S, Wang P, Burden of non-alcoholic fatty liver disease-related cirrhosis and other chronic liver diseases from 1990 to 2019 in China and disease burden trend prediction to 2030. Chin Med J (Engl). 2024;137(18):2251–2253. 39030075 10.1097/CM9.0000000000003211 PMC11407810

[5] Hoogendijk EO, Afilalo J, Ensrud KE, Kowal P, Onder G, Fried LP. Frailty: implications for clinical practice and public health. Lancet. 2019;394(10206):1365–1375. 31609228 10.1016/S0140-6736(19)31786-6

[6] Lai JC, Tandon P, Bernal W, Malnutrition, Frailty, and Sarcopenia in Patients With Cirrhosis: 2021 Practice Guidance by the American Association for the Study of Liver Diseases. Hepatology. 2021;74(3):1611–1644. 34233031 10.1002/hep.32049 PMC9134787

[7] Lai JC, Shui AM, Duarte-Rojo A, Association of Frailty With Health-Related Quality of Life in Liver Transplant Recipients. JAMA Surg. 2023;158(2):130–138. 36515937 10.1001/jamasurg.2022.6387 PMC9856900

[8] Álvarez-Bustos A, Carnicero-Carreño JA, Sanchez-Sanchez JL, Garcia-Garcia FJ, Alonso-Bouzón C, Rodríguez-Mañas L. Associations between frailty trajectories and frailty status and adverse outcomes in community-dwelling older adults. J Cachexia Sarcopenia Muscle. 2022;13(1):230–239. 34951157 10.1002/jcsm.12888 PMC8818602

[9] Yang H, Ou F, Chang Q, Physical frailty, genetic predisposition, and the risks of severe non-alcoholic fatty liver disease and cirrhosis: a cohort study. J Cachexia Sarcopenia Muscle. 2024;15(4):1491–1500. 38887910 10.1002/jcsm.13506 PMC11294048

[10] Lei W, Hui L, Runkuo Z, Association Between Frailty and Chronic Liver Disease Among Middle-Aged and Older Adults in China: A Nationwide Cohort Study Based on the CHARLS. Geriatr Gerontol Int. 2026;26(5):e70501. 42053035 10.1111/ggi.70501

[11] Deng MG, Liu F, Liang Y, Wang K, Nie JQ, Liu J. Association between frailty and depression: A bidirectional Mendelian randomization study. Sci Adv. 2023;9(38):eadi3902. 37729413 10.1126/sciadv.adi3902 PMC10511184

[12] Jiang R, Noble S, Rosenblatt M, The brain structure, inflammatory, and genetic mechanisms mediate the association between physical frailty and depression. Nat Commun. 2024;15(1):4411. Published 2024 May 23. 38782943 10.1038/s41467-024-48827-8 PMC11116547

[13] Wang H, Zhao S, Wang X, Liu X. Quantifying depression and the risk of chronic liver diseases: results from a large-scale longitudinal cohort study. Front Public Health. 2025;13:1584211. Published 2025 Sep 26. 41080849 10.3389/fpubh.2025.1584211 PMC12511029

[14] Kim D, Dennis BB, Cholankeril G, Ahmed A. Association between depression and metabolic dysfunction-associated fatty liver disease/significant fibrosis. J Affect Disord. 2023;329:184–191. 36841305 10.1016/j.jad.2023.02.101

[15] Zhao Y, Hu Y, Smith JP, Strauss J, Yang G. Cohort profile: the China Health and Retirement Longitudinal Study (CHARLS). Int J Epidemiol. 2014;43(1):61–68. 23243115 10.1093/ije/dys203 PMC3937970

[16] Searle SD, Mitnitski A, Gahbauer EA, Gill TM, Rockwood K. A standard procedure for creating a frailty index. BMC Geriatr. 2008;8:24. Published 2008 Sep 30. 18826625 10.1186/1471-2318-8-24 PMC2573877

[17] Searle SD, Rockwood K. What proportion of older adults in hospital are frail?. Lancet. 2018;391(10132):1751–1752. 29706363 10.1016/S0140-6736(18)30907-3

[18] Tang WZ, Deng BY, Cai QY, The trajectory of frailty index and its association with rapid decline in kidney function and the incidence of chronic kidney disease: evidence from the China health and retirement longitudinal study. Aging Clin Exp Res. 2025;37(1):238. Published 2025 Aug 2. 40751850 10.1007/s40520-025-03146-w PMC12317918

[19] Yang X, Ma J, Li H. Trajectories of depressive symptoms and risk of chronic liver disease: evidence from CHARLS. BMC Gastroenterol. 2025;25(1):338. Published 2025 May 7. 40335900 10.1186/s12876-025-03943-7 PMC12057118

[20] Li W, Zhou Y, Li Q, Wang D. The relationship between dyslipidemia and chronic liver disease, with the mediating role of depressive symptoms. Front Public Health. 2025;13:1581622. Published 2025 Aug 25. 40927334 10.3389/fpubh.2025.1581622 PMC12414773

[21] Liu Q, Huang Y, Wang B, Joint trajectories of pain, depression and frailty and associations with adverse outcomes among community-dwelling older adults: A longitudinal study. Geriatr Nurs. 2024;59:26–32. 38981205 10.1016/j.gerinurse.2024.06.039

[22] Chai S, Zhao D, Gao T, The relationship between handgrip strength and cognitive function among older adults in China: Functional limitation plays a mediating role. J Affect Disord. 2024;347:144–149. 37992778 10.1016/j.jad.2023.11.056

[23] Zhong Q, Zhou R, Huang YN, Frailty and risk of metabolic dysfunction-associated steatotic liver disease and other chronic liver diseases. J Hepatol. 2025;82(3):427–437. 39218228 10.1016/j.jhep.2024.08.024

[24] Zhang F, Xiong YJ, Meng XD, Lv T, Yang DJ. Joint association of frailty and depression with new-onset digestive disease among elderly Chinese population. Front Nutr. 10.3389/fnut.2025.1590194 PMC12336231 40791236

[25] Le Bert N, Fisicaro P. Enolase: a metabolic checkpoint behind diverse exhaustion stages of CD8+ T cells in chronic HBV and HCV. Gut. 2023;72(10):1814–1815. 37673656 10.1136/gutjnl-2023-330541

[26] Perazza LR, Brown-Borg HM, Thompson LV. Physiological Systems in Promoting Frailty. Compr Physiol. 2022;12(3):3575–3620. Published 2022 Apr 26. 35578945 10.1002/cphy.c210034 PMC9531553

[27] Endo-Umeda K, Makishima M. Liver X Receptors Regulate Cholesterol Metabolism and Immunity in Hepatic Nonparenchymal Cells. Int J Mol Sci. 2019;20(20):5045. Published 2019 Oct 11. 31614590 10.3390/ijms20205045 PMC6834202

[28] Losasso MR, Parussolo MLC, Oliveira Silva A, Unraveling the Metabolic Pathways Between Metabolic-Associated Fatty Liver Disease (MAFLD) and Sarcopenia. Int J Mol Sci. 2025;26(10):4673. Published 2025 May 14. 40429815 10.3390/ijms26104673 PMC12111209

[29] Cai C, Song X, Chen Y, Chen X, Yu C. Relationship between relative skeletal muscle mass and nonalcoholic fatty liver disease: a systematic review and meta-analysis. Hepatol Int. 2020;14(1):115–126. 31290072 10.1007/s12072-019-09964-1 PMC6994447

[30] Gómez de Las Heras MM, Carrasco E, Pérez-Manrique M, CD4 T cell therapy counteracts inflammaging and senescence by preserving gut barrier integrity. Sci Immunol. 2025;10(110):eadv0985. 40749035 10.1126/sciimmunol.adv0985 PMC7618201

[31] Liu S, Yang X. Intestinal flora plays a role in the progression of hepatitis-cirrhosis-liver cancer. Front Cell Infect Microbiol. 2023;13:1140126. Published 2023 Mar 9. 36968098 10.3389/fcimb.2023.1140126 PMC10034054

[32] Qie R, Huang H, Sun P, Combined healthy lifestyles and risk of depressive symptoms: A baseline survey in China. J Affect Disord. 2024;363:152–160. 39038619 10.1016/j.jad.2024.07.134

[33] Wu W, Ma W, Yuan S, Associations of Unhealthy Lifestyle and Nonalcoholic Fatty Liver Disease With Cardiovascular Healthy Outcomes. J Am Heart Assoc. 2023;12(23):e031440. 38014686 10.1161/JAHA.123.031440 PMC10727317

[34] Sang N, Liu RC, Zhang MH, Changes in frailty and depressive symptoms among middle-aged and older Chinese people: a nationwide cohort study. BMC Public Health. 2024;24(1):301. Published 2024 Jan 25. 38273230 10.1186/s12889-024-17824-3 PMC10811919

[35] Zeng J, Lai X, Wang S, Association of depressive symptoms with chronic liver disease among middle-aged and older adults in China. Front Psychiatry. 2023;14:1273754. Published 2023 Oct 24. 37941967 10.3389/fpsyt.2023.1273754 PMC10628464

